# Environmental and Genetic Preconditioning for Long-Term Anoxia Responses Requires AMPK in *Caenorhabditis elegans*

**DOI:** 10.1371/journal.pone.0016790

**Published:** 2011-02-03

**Authors:** Bobby L. LaRue, Pamela A. Padilla

**Affiliations:** Department of Biological Sciences, University of North Texas, Denton, Texas, United States of America; Brown University, United States of America

## Abstract

**Background:**

Preconditioning environments or therapeutics, to suppress the cellular damage associated with severe oxygen deprivation, is of interest to our understanding of diseases associated with oxygen deprivation. Wildtype *C. elegans* exposed to anoxia enter into a state of suspended animation in which energy-requiring processes reversibly arrest. *C. elegans* at all developmental stages survive 24-hours of anoxia exposure however, the ability of adult hermaphrodites to survive three days of anoxia significantly decreases. Mutations in the insulin-like signaling receptor (*daf-2*) and LIN-12/Notch (*glp-1*) lead to an enhanced long-term anoxia survival phenotype.

**Methodology/Principal Findings:**

In this study we show that the combined growth environment of 25°C and a diet of HT115 *E. coli* will precondition adult hermaphrodites to survive long-term anoxia; many of these survivors have normal movement after anoxia treatment. Animals fed the drug metformin, which induces a dietary-restriction like state in animals and activates AMPK in mammalian cell culture, have a higher survival rate when exposed to long-term anoxia. Mutations in genes encoding components of AMPK (*aak-2*, *aakb-1*, *aakb-2*, *aakg-2*) suppress the environmentally and genetically induced long-term anoxia survival phenotype. We further determine that there is a correlation between the animals that survive long-term anoxia and increased levels of carminic acid staining, which is a fluorescent dye that incorporates in with carbohydrates such as glycogen.

**Conclusions/Significance:**

We conclude that small changes in growth conditions such as increased temperature and food source can influence the physiology of the animal thus affecting the responses to stress such as anoxia. Furthermore, this supports the idea that metformin should be further investigated as a therapeutic tool for treatment of oxygen-deprived tissues. Finally, the capacity for an animal to survive long bouts of severe oxygen deprivation is likely dependent on specific subunits of the heterotrimeric protein AMPK and energy stores such as carbohydrates.

## Introduction

Oxygen deprivation is central to many life-threatening human health issues including blood loss, pulmonary disorders, stroke and myocardial infarction. Human organs such as the heart and brain are particularly vulnerable to oxygen and nutrient deprivation caused by blood loss or a decrease in blood delivery [1], [2]. Clinically, the goal is to quickly reestablish blood flow to ischemic areas and thus minimize the cellular damage induced by ischemia; however, this reperfusion can also cause cellular damage. Preconditioning environments or therapeutics, to suppress the cellular or tissue damage associated with severe oxygen deprivation, are being heavily investigated [3], [4]. Preconditioning is a protective technique in which organisms, prior to ischemic bouts, are exposed to a specific environment or a cellular change is induced genetically or pharmacologically. For example, ischemic preconditioning, a brief episode(s) of non-lethal ischemia prior to coronary artery occlusion, has been shown to reduce infarct size [5], [6], [7]. The mechanism for ischemic preconditioning in mammals is not completely understood but it does involve several signal transduction pathways including phsphatidylinositol-3-kinase (PI3K)-AKT, extracellular signal regulated kinase (ERK1/2) and hypoxia inducing factor (HIF-1) [8], [9], [10]. An understanding of the molecular changes that influence an organisms' ability to survive severe oxygen deprivation is of interest to understanding human health related injuries induced by ischemia and reperfusion. Furthermore, finding a pharmacological means to render cells resistant to oxygen deprivation is of significant interest.

There are a number of animals that are tolerant to oxygen deprivation [11], [12]. Often these animals have evolved ways to maintain tissue and cellular homeostasis when challenged with oxygen deprivation. For example, energy-requiring processes such as protein translation, ion transport, development, or cell cycle progression are slowed or arrested to conserve energy [13], [14], [15], [16], [17]. Another strategy includes physiological alterations leading to an organism's ability to survive when challenged with frequent exposures to oxygen deprivation [18]. For example, in diving seals there is an increase in the oxygen-binding molecule myoglobin in specific tissues and a shunting of oxygen and nutrients to energetically demanding tissues such as the brain [19], [20]. Additionally, there are highly conserved responses to oxygen deprivation such as those that involve the transcription factor Hypoxia Inducing Factor (HIF-1) [21]. Changes in gene expression induced by HIF-1 enable organisms, including humans, to respond to hypoxic environments by inducing the expression of specific genes that are important for oxygen homeostasis. Much is known about the HIF-1 signaling pathway, however the mechanisms regulating non-HIF-1 related responses to oxygen deprivation in hypoxia-tolerant organisms is not understood.

Studies using genetic model systems such as *Caenorhabditis elegans* and *Drosophila melanogaster* have led to a greater understanding of mechanisms important for oxygen deprivation response and survival [15], [22], [23], [24]. *C. elegans*, as a soil nematode, has evolved mechanism to sense various oxygen tensions [25], [26], [27]. Furthermore, genetic alleles have been identified that either enhance or suppress oxygen deprivation survival [28], [29], [30]. For example, mutations in the insulin/IGF-1 receptor-like gene *daf-2* protect animals from severe hypoxia [31]. Wildtype *C. elegans* exposed to anoxia (growth temperature of 20°C), enter into a state of suspended animation in which energy requiring processes, such as development, cell division, ovulation and egg laying, reversible arrest [32], [33], [34], [35], [36]. Animals at all developmental stages survive 24-hours of anoxia exposure however, the ability of adult hermaphrodites to survive long-term anoxia (three or more days of anoxia exposure) significantly decreases. Mutations in *daf-2* enhance anoxia survival; this survival is dependent on the transcription factor DAF-16 and the glycolytic enzyme glyceraldehyde-3-phosphate dehydrogenase GPD-2/3. Furthermore, genetic mutations that induce sterility or decrease ovulation (Example: *glp-1(q158)*, *fog-2(q71)*) lead to the hermaphrodites ability to survive long-term anoxia; the mechanism for this enhanced anoxia survival phenotype is not understood [37], [38]. It is known that the HIF-1 signaling pathway is not required for anoxia survival in *C. elegans* [32].

Genetic studies in *C. elegans* have demonstrated that there is an overlap between some of the mechanisms regulating lifespan, energy homeostasis, dauer formation and stress responses including oxygen deprivation [39], [40], [41]. First, the role the *daf-2/daf-16* signaling pathway has in dauer formation and aging is well understood but it is not yet clear which components of this pathway are important for anoxia responses and survival [38], [42], [43]. Second, it has also been determined that reproduction influences both aging and stress resistance [37], [44]. For example, animals that are sterile due to a mutation in *glp-1* (LIN-12/Notch) are long lived, yet the longevity phenotype is not merely due to sterility as laser ablation of somatic and germ cells in wildtype animals does not lead to longevity [45]. The *glp-1* mutant animals are also able to survive long-term anoxia [37]. It is not yet clear if the mechanisms regulating longevity and anoxia survival in the *glp-1* mutant are identical or overlap. Finally, it is known that there is a relationship between an organism's energy level and aging. For example, a limit of energy by dietary restriction is known to extend life span in many organisms; the genetic regulation of this is being worked out in various model systems including *C. elegans* [46], [47]. In eukaryotic cells, a sensor of energy levels is the serine/threonine AMP-activated protein kinase (AMPK). AMPK, is a heterotrimeric complex consisting of a catalytic subunit (α) and two regulatory subunits (β,γ); several genes encode these subunits [48]. AMPK is activated by AMP and inhibited by ATP levels; activated AMPK switches off ATP consuming processes and switches on ATP producing pathways such as glycolysis and fatty acid oxidation. In *C. elegans* it was shown that the AMP/ATP ratio increases with age. Furthermore, the a-subunit of AMPK, encoded by *aak-2*, functions in lifespan, dauer formation and stress responses [49], [50], [51], [52]. Over expression of AAK-2 increases longevity and *aak-2* functions downstream of *daf-2*-mediated insulin signaling, to positively regulate adult lifespan [50]. It is not yet understood if AMPK has a role in anoxia responses and survival.

In this study we show that the combined environment of 25°C growth condition and the feeding of HT115 *E. coli* strain will precondition adult hermaphrodites so that they have an enhanced long-term anoxia survival phenotype. This enhanced long-term anoxia phenotype is defined here as the ability of animals to survive three or four days of anoxia and that many of the survivors have normal movement after anoxia treatment. Previously we showed that mutations in the insulin-like signaling pathway (*daf-2*) and LIN-12/Notch (*glp-1*) lead to an enhance anoxia survival phenotype [37], [38]. Here we show mutations in genes encoding components of AMPK will suppress environmentally-induced (temperature and food source) and genetically-induced (*daf-2* or *glp-1* mutants) long-term anoxia viability. Animals fed the drug metformin, which induces a dietary-restriction like state in animals and activates AMPK in mammalian cell culture [53], have a higher survival rate when exposed to long-term anoxia. We further determine that there is a correlation between the animals that survive long-term anoxia and increased levels of carminic acid, which is a fluorescent dye that incorporates in with carbohydrates such as glycogen. Together, these results show that environmental effects such as *E. coli* food source and growth temperature, and pharmacological agents such as metformin, can influence the ability of *C. elegans* to survive anoxia. Furthermore, specific components of the AMPK, and carbohydrates stores are likely to be involved with responses to anoxia.

## Results

### Temperature and food source preconditions for long-term anoxia survival

**Figure 1:**
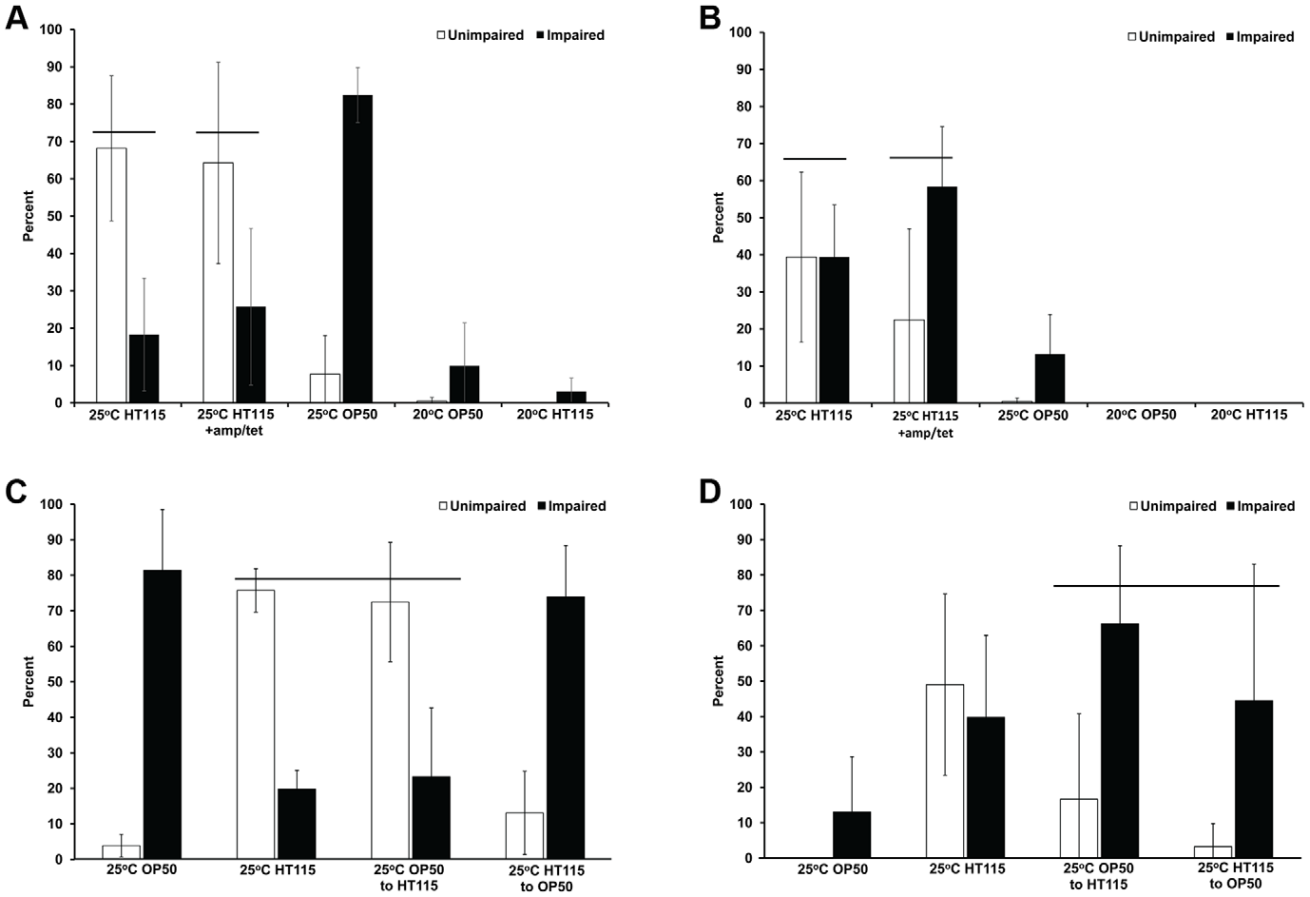
Temperature and *E. coli* food source preconditions for long-term anoxia survival. N2 adult hermaphrodites were raised at either 25°C or 20°C, fed either the OP50 or HT115 *E. coli* strain, exposed to either three days (A) or four days of anoxia (B). The survivors were examined for an unimpaired or impaired phenotype. The plates that contained antibiotics and temperature in which animals were grown on is noted as such. Animals grown at 25°C have a higher survival rate in comparison to those grown at 20°C (p<.05, Student's T-test) (A, B). Animals grown at 25°C and fed HT115 *E. coli* strain, in comparison to those raised at 25°C and fed OP50 *E. coli*, have a significantly higher unimpaired phenotype after long-term anoxia exposure (A). Line denotes statistically significant groups (α = .05, SNK multiple range test) (A, B). Food switching experiments involve the feeding of one food source during development and then transferred to the other food source prior to three or four days of anoxia exposure (C, D); survivors were examined for an unimpaired or impaired phenotype. Animals fed OP50 during development and then transferred to the HT115 strain have a significantly higher unimpaired phenotype in comparison to those animals fed HT115 during development and then transferred to OP50 prior to anoxia exposure or to those animals only fed OP50. (C). Animals that were raised on HT115 or transferred to HT115 had a significantly higher survival rate then those only raised on OP50. Line denotes statistically significant groups, α = .05, SNK multiple range test (C, D). For all experiments, the total number of animals assayed is N>175 from four independent experiments; error bar represents standard deviation.

It is known that some environments will precondition animals to better survive oxygen deprivation; however, often the mechanism regulating preconditioning is not understood. Previously, we showed that *C. elegans* hermaphrodites grown at 20°C and exposed to one day of anoxia (at 20°C) have a >90% survival rate, yet animals exposed to long-term anoxia (defined here as three or more days of anoxia exposure) have a drastic reduction in viability [32], [38]. To determine if the temperature an animal develops at will influence the anoxia survival phenotype we let animals develop from L1 larvae to 1-day old adulthood at 25°C, instead of the common 20°C growth condition, and then exposed them to long-term anoxia exposure. In these assays, after anoxia exposure the animals were examined for viability (See Table S1) and survivors were further scored as having either an “unimpaired” or “impaired” phenotype. That is, an animal was scored as unimpaired if it did not have any visible defects in motility or tissue morphology; an animal was scored as impaired if it had defects in motility or tissue morphology. The classification of anoxia-survivors to either of these two categories helped delineate between animals that were either more resistant or sensitive to long-term anoxia and if tissue function may have been compromised by the anoxia treatment. We determined that animals grown to adulthood at 25°C, as opposed to 20°C, had a significantly higher long-term anoxia survival rate (Figure 1A, 1B). The animals grown at 25°C and fed the common *E. coli* strain OP50, survived long-term anoxia yet many of the animals were impaired, as indicated by visible defects in motility or tissue morphology (Figure 1A, Video S1). Animals grown on the HT115 *E. coli* strain at 25°C also survived long-term anoxia and surprisingly many of the survivors were unimpaired (Figure 1A, 1B, Video S2). The animals fed HT115 and grown at 25°C did have a decrease in survival rate when exposed to four days of anoxia in comparison to three days of anoxia, however they still survived at a significantly higher rate in comparison to animals grown at 20°C. The HT115 *E. coli* strain is used in RNA interference studies [54]; it contains the L4440 plasmid (selected for by ampicillin) and lacks the dsRNA-specific endonuclease RNaseIII (selected for by tetracycline). We found that the anoxia survival rates of animals fed HT115 were not affected by the presence or absence of the antibiotics (Figure 1A, 1B). These results suggest that the HT115 strain diet, in comparison to the OP50 strain, has a different effect on the physiology of *C. elegans* grown at 25°C.

We conducted food-switching experiments to determine if enhanced anoxia survival only occurred if the animals were fed HT115 throughout their development or if feeding the HT115 strain prior to anoxia exposure is sufficient for an enhanced anoxia survival phenotype. Animals were raised to adulthood on either HT115 or OP50 and then transferred to the alternate *E. coli* strain and placed into three or four days of anoxia. We found that animals fed OP50 throughout development and then transferred to HT115 (OP50 to HT115) survived long-term anoxia (3 days) and the number of unimpaired survivors was significantly higher in comparison to the animals only fed OP50 (Figure 1C). The animals fed HT115 throughout development and then transferred to OP50 (HT115 to OP50) also survived anoxia; however, these animals had a significantly higher number of impaired animals in comparison to those only fed HT115 (Figure 1C). Animals that were raised on or transferred to HT115 prior to four days of anoxia exposure had a significantly higher survival rate then those only fed OP50 (Figure 1D, Table S1). We know from previous studies that animals exposed to anoxia enter into a reversible state of suspended animation in which motility will arrest [32]. However, wildtype adult animals remain motile for up to 8 hours after anoxia exposure before arresting movement; these animals likely eat before they enter into suspended animation [38]. Together, these data suggest that the combination of animals being grown at 25°C and fed the HT115 *E. coli* strain will precondition animals so that they have an enhanced long-term anoxia survival phenotype.

**Figure 2:**
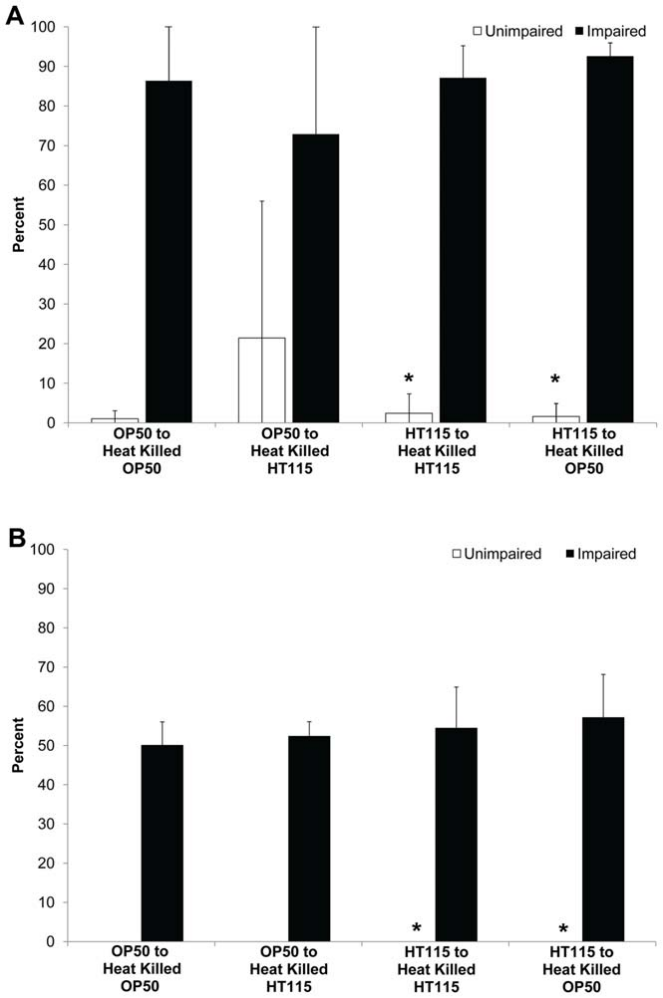
Animals fed heat-killed HT115 have an impaired phenotype after anoxia treatment. N2 adult hermaphrodites were fed either the OP50 or HT115 *E. coli* strain, raised at 25°C, transferred to heat-killed OP50 or HT115 and exposed to either three days (A) or four days of anoxia (B). Food switching experiments involve the feeding of one food source (live bacteria) during development and then transferred to the other food source (head killed bacteria) prior to three or four days of anoxia exposure. Transfer of animal to heat killed HT115 resulted in a significant decrease in the unimpaired phenotype (*). For all experiments, the total number of animals assayed is N>168 from four independent experiments; error bar represents standard deviation. (p<.05, Student's T-test).

To determine if live OP50 produces a toxic metabolite that leads to an impaired phenotype after anoxia treatment or alternatively if live HT115 provides a beneficial metabolite prior to or during anoxia treatment we grew worms on live HT115 or OP50 and then transferred worms to heat killed OP50 or HT115 prior to anoxia treatment. We determined that eating either strain of heat-killed bacteria did not decrease the survival rate when exposed to long-term anoxia (Figure 2, Table S1). This suggests that live OP50 does not leak some toxic metabolite that increases the unimpaired phenotype. Animals that were grown on HT115 and then transferred to heat killed HT115 no longer had the unimpaired phenotype. Combined, these data shown in Figure 1 and 2 indicate that animals that eat live HT115 bacteria prior to anoxia treatment have an increased unimpaired phenotype relative to those animals on other diets.

### Suppression of the insulin signaling pathway and AMPK suppresses the environmentally induced, anoxia-preconditioning affect

**Figure 3:**
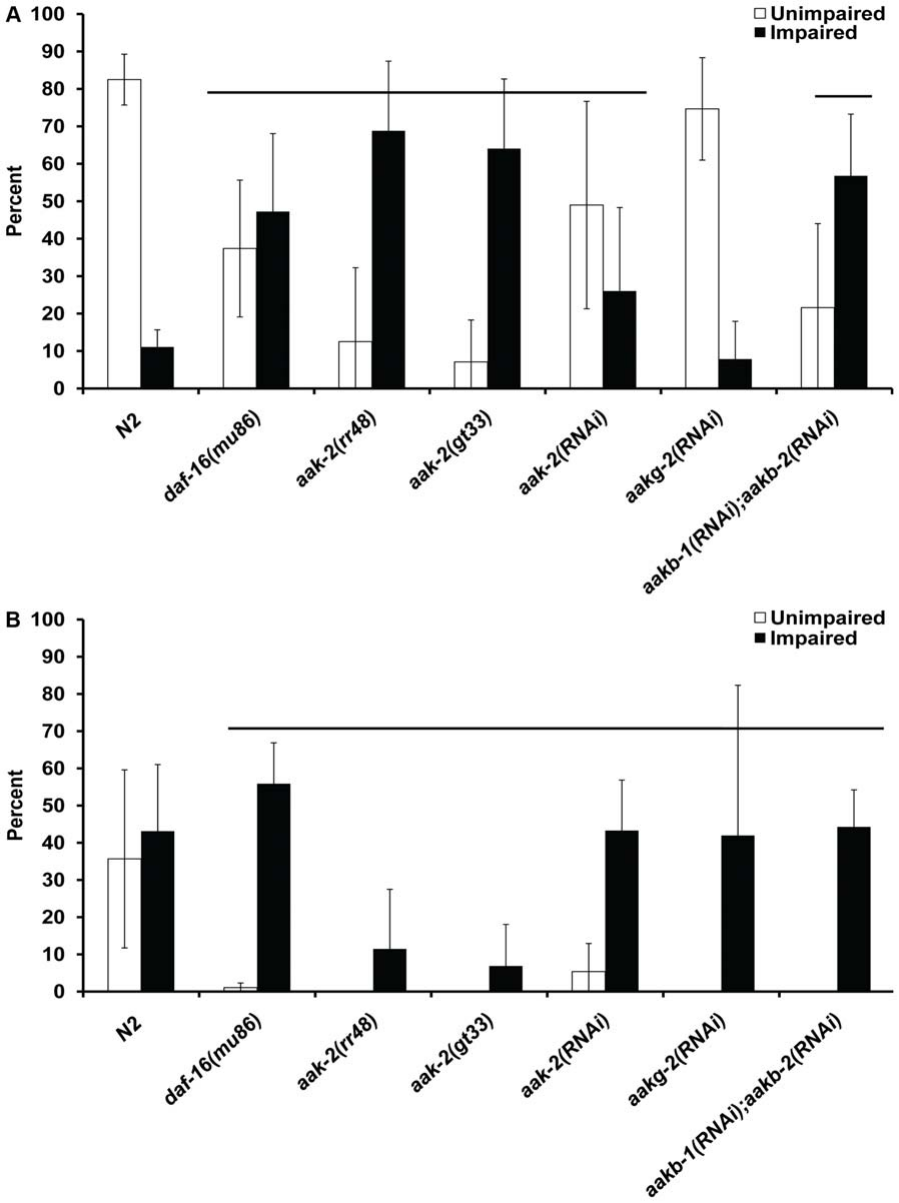
Reduction in *aak-2* or *daf-16* function suppresses the long-term anoxia survival preconditioning induced by temperature and *E. coli* food source. Adult hermaphrodites, of specified genotype or RNAi experiment, were raised at 25°C (HT115 *E. coli* food source) and exposed to either three days (A) or four days (B) of anoxia; survivors were examined for an unimpaired or impaired phenotype. Line denotes groups with a significant decrease in the number of animals with an unimpaired phenotype in comparison to N2 control (p<. 05, Dunnett's Multiple Range test) (A). The *aak-2* mutants exposed to four days of anoxia had a significant decrease in survival rate in comparison to control (B). Line denotes groups with a significant decreae in the number of animals with an unimpaired phenotype in comparison to N2 control (p<. 05, Dunnett's Multiple Range test). For all experiments, the total number of animals assayed is N>180 from four independent experiments; error bar represents standard deviation.

It is possible that the environment in which the animal is exposed to (growth at 25°C and HT115 *E. coli* diet) will precondition for enhanced anoxia survival by altering the physiology of the animal; this altered physiology may be dependent upon specific genetic pathways. There are several lines of evidence that led us to further investigate if signaling pathways involved with aging (insulin-like signaling, LIN-12/Notch and AMP activated kinase (AMPK)) have a role in long-term anoxia survival. First, genetic changes in the insulin-like signaling pathway or LIN-12/Notch signaling pathway increases long-term anoxia survival; the *daf-2(e1370)* and *glp-1(q158)* animals are long lived and have a high long-term anoxia survival phenotype [37], [38], [45], [55]. Second, the ratio of AMP/ATP increases in wildtype *C. elegans* exposed to anoxia [32]. Others have shown an increase in the AMP/ATP ratio in mammals activates AMPK [56], [57] and AMPK is thought to have a role in longevity [50]. Finally, there is evidence that the ability to store carbohydrates such as glycogen correlates with survival of stresses including anoxia [35]. We took a genetic approach to determine if the enhanced long-term anoxia survival phenotype of animals grown at 25°C on HT115 food could be suppressed by mutations in either an AMPK component or the FOXO transcription factor *daf-16*. The *daf-16* mutants exposed to three days of anoxia did not show a significant difference in overall survival when compared to that of control (Figure 3A, Table S2). However, those exposed to three or four days of anoxia had a significant decrease in animals with an unimpaired phenotype (Figure 3).

AMPK, a conserved sensor of energy levels, is a heterotrimeric protein composed of the catalytic αsubunit, and the regulatory β and γ subunits; *C. elegans* have two α-subunits, two β-subunits and five predicted γ-subunits [58]. We tested if subunits of AMPK, for which we have either mutants or RNAi food available, are required for environmental preconditioning for enhanced anoxia survival phenotype (Table S2). The *aak-2* gene encodes one of two catalytic α-subunits of AMPK. To determine if mutations in *aak-2* activity suppressed the enhanced anoxia phenotype in preconditioned animals, we used two *aak-2* alleles (*gt33* which has a 606 bp deletion in exon 3 and a presumed molecular null, and *rr48*, which has a point mutation predicted to disrupt the catalytic activity) [51], [59]. The overall survival rate after anoxia treatment was not altered in *aak-2* mutant animals exposed to three days of anoxia (Supplemental Table S2). However, there is a significant decrease in the number of *aak-2* mutant animals with an unimpaired phenotype in comparison to wildtype animals (Figure 3A). The *aak-2* mutants exposed to four days of anoxia had a significant decrease in survival rate in comparison to control (Table S2, Figure 3B). The *aakg-2(RNAi)* animals exposed to anoxia for four days had a decrease in the number of unimpaired animals and overall survivors (Figure 3B, Table S2). We did not observe a suppression of the enhanced anoxia survival phenotype with knockdown of other AMPK subunits (Table S2). We did not observe a phenotype associated with individually knocking down *aakb-1* and *aakb-2.* However, the *aakb-1(RNAi);aakb-2(RNAi)* animals exposed to three or four days of anoxia had a reduction in the number of animals with an unimpaired phenotype and overall lower survival rate. These data together suggest that *aak-2*, *aakb-1*, *aakb-2* and *aakg-2* have a role in maintenance of tissue during anoxia exposure.

Wildtype *C. elegans* grown at 20°C and exposed to long-term anoxia have a severe reduction in viability (Figure 1A). Previously we showed that the *daf-2(e1370)* and *glp-1(q158)* animals, raised in similar environments prior to anoxia exposure, are among the most viable anoxia survival mutants we have identified to date. In these previous studies a mutation in *daf-16* was able to suppress the long-term anoxia survival phenotype in *daf-2(e1370)* animals but *daf-16(RNAi)* did not suppress the enhanced anoxia phenotype observed in *glp-1(q158)* animals [37], [38]. To further investigate the role AMPK has in the enhanced-anoxia survival phenotype we tested if components of the AMPK could suppress anoxia viability in *daf-2(e1370)* and *glp-1(e2141)* animals. Note that experiments involving the *daf-2(e1370)* animal are done at 20°C because of the 25°C dauer constitutive phenotype. The *glp-1(e2141)* mutant is a temperature sensitive mutant in which sterility is observed when grown at 25°C and thus these experiments are conducted at 25°C.

**Figure 4:**
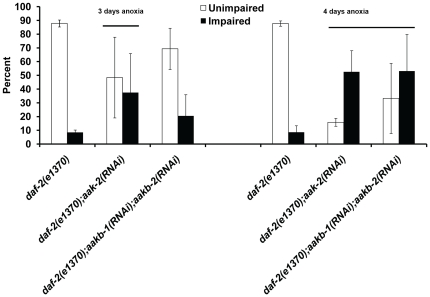
Suppression of *daf-2(e1370)* enhanced long-term anoxia phenotype. Adult hermaphrodites of the given genotype were fed HT115 *E. coli* strain or specified RNAi food and exposed to either three days (A) or four days of anoxia (B). The survivors were examined for an unimpaired or impaired phenotype. Line denotes groups with a significant decrease in the number of animals with an unimpaired phenotype in comparison to *daf-2(e1370)* (p<.05 Student's paired one tailed-T-test). For all experiments, the total number of animals assayed is N>180 from four independent experiments; error bar represents standard deviation.

**Figure 5:**
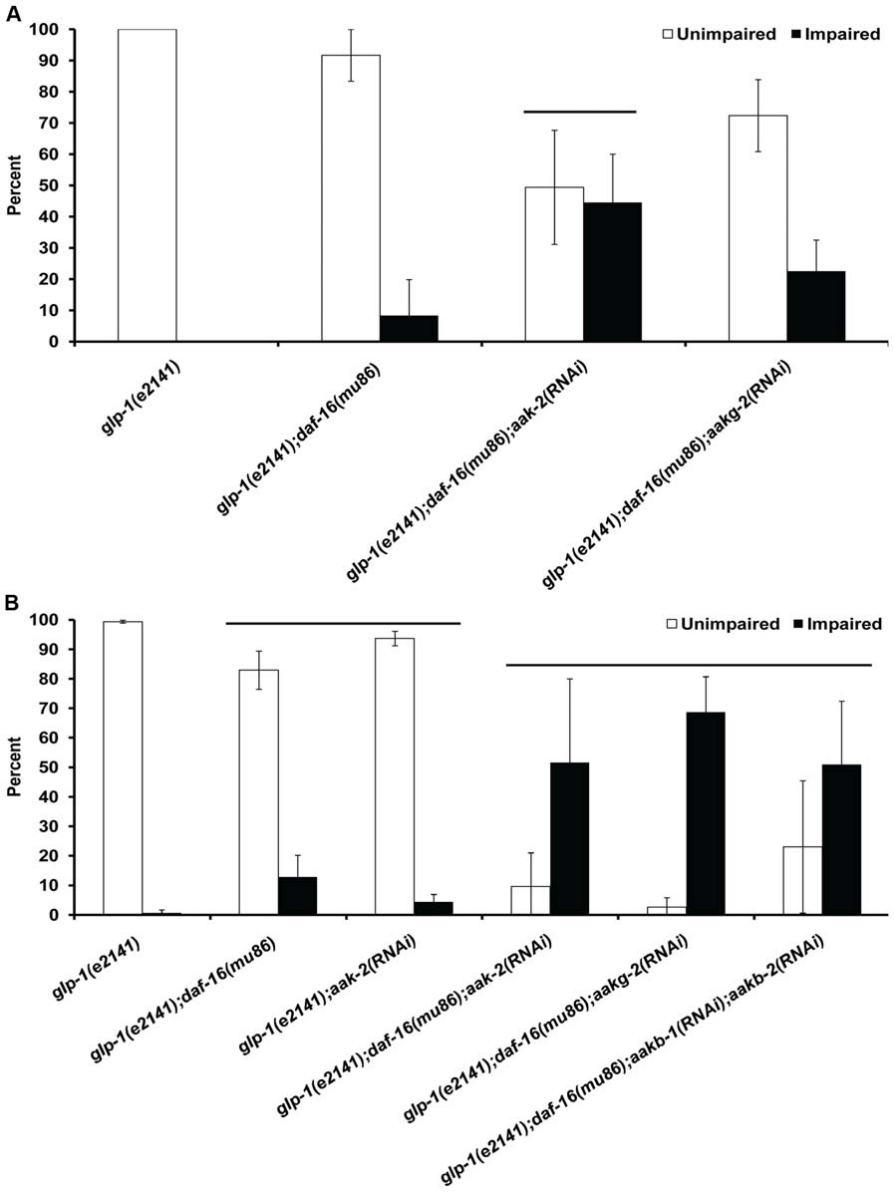
Suppression of *glp-1(e2141)* enhanced long-term anoxia phenotype. Adult hermaphrodites of the given genotype were fed HT115 *E. coli* strain or specified RNAi food and exposed to either three days (A) or four days of anoxia (B). The survivors were examined for an unimpaired or impaired phenotype. Line denotes groups with a significant decrease in the number of animals with an unimpaired phenotype in comparison to *glp-1(e2141)* (p<.05 Student's paired one tailed-T-test). For all experiments, the total number of animals assayed is N>180 from four independent experiments; error bar represents standard deviation.

The *daf-2(e1370)* and *glp-1(e2141)* animals fed HT115 food prior to three or four days of anoxia exposure have a high long-term anoxia survival phenotype in which the majority of the animals were unimpaired after treatment (Figures 4 and 5, Table S3 and S4). The *daf-2(e1370);aak-2(RNAi)* animals exposed to either three or four days of anoxia had a significant decrease in the unimpaired phenotype in comparison to *daf-2(e1370)* animals (Figure 4); the survival rate for the *daf-2(e1370);aak-2(RNAi)* animals decreased when exposed to four days of anoxia (Table S3). The unimpaired phenotype observed in *daf-2(e1370)* animals was also suppressed when *aakb-1* and *aakb-2* was reduced by RNAi, however the overall survival rate was not significantly reduced (Figure 4, Supplemental Table S3).

In the case of *glp-1(e2141)* animals, a reduction of function of either *daf-16* or *aak-2* alone did not significantly reduce the viability or unimpaired phenotype after three days of anoxia treatment. However, there was a reduction in the number of unimpaired animals after four days of anoxia treatment (Figure 5B). If both *daf-16* and *aak-2* function was reduced simultaneously the survival rate was still high (Table S5) however the number of animals that were unimpaired was reduced after anoxia treatment (Figure 5). The number of animals with an unimpaired phenotype, after four days of anoxia exposure, was significantly suppressed by RNAi of *aak-2*, *aakg-2* or *aakb-1/aakb-2* in the background of the *daf-16(mu86)* null mutation. These data suggest that DAF-16 and specific components of AMPK (*aak-2*, *aakg-2*, *aakb-1*, *aakb-2*) function in parallel to enhance anoxia survival in *glp-1* mutant animals.

### Metformin preconditions for long-term anoxia survival

**Figure 6:**
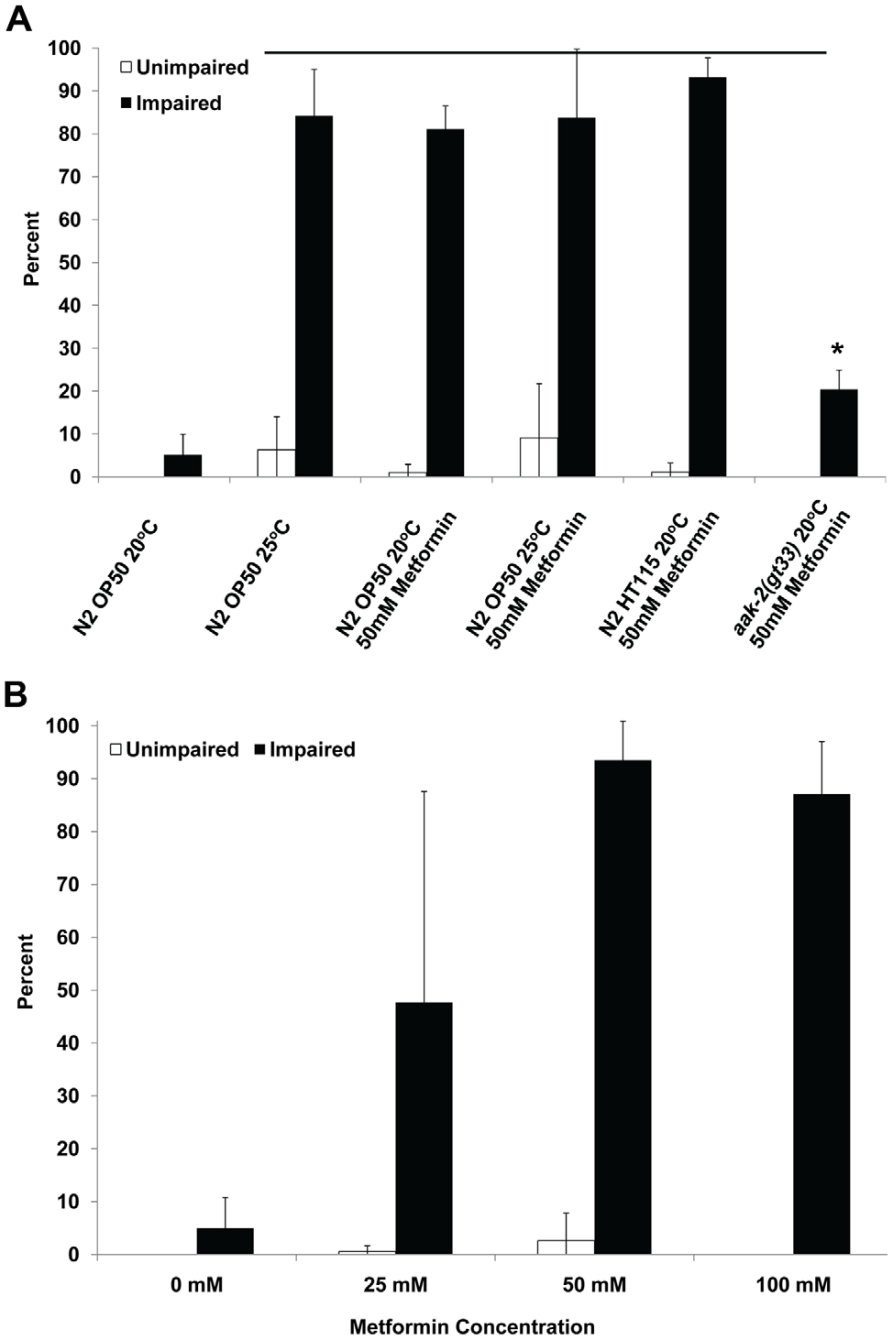
Wild-type animals fed metformin survive long-term anoxia. N2 adult hermaphrodites were raised in the specified conditions from L1 larvae to one-day old adult and then exposed to three days of anoxia. The survivors were examined for an unimpaired or impaired phenotype. Line indicates a statistically significant increase in overall survival compared to animals grown at 20°C on OP50 bacteria in the absence of 50 mM Metformin (p<.05 Student's paired one-tailed T-test). The long-term anoxia survival rate of *aak-2(gt33)* fed metformin was significantly less in comparison to wildtype animals fed metformin (*). For all experiments the total number of animals assays is N>180 from four independent experiments; error bars indicate standard deviation.

Metformin is a drug that induces a dietary restriction-like state in animals, reduces blood glucose levels and is used to treat type-2 diabetes. Mammalian studies show that metformin works in part by activation of AMPK. In *C. elegans* metformin extends lifespan and induces a dietary restriction-like state and oxidative stress response; the lifespan benefits of metformin is dependent on AAK-2, its upstream kinase LKB1/PAR-4 and transcription factor SKN-1. To determine if metformin can induce an enhanced anoxia survival phenotype in *C. elegans*, larvae were raised at the non-preconditioning temperature of 20°C on NGM plates containing various levels of metformin and seeded with OP50; adult animals were then exposed to three days of anoxia and examined for an impaired or unimpaired phenotype (Figure 6). The animals exposed to 25, 50 or 100 mM of metformin had a significantly higher survival rate in comparison to control (Figure 6B, Table S6). However, the majority of these animals had an impaired phenotype. Animals that were raised on 250 or 500 mM metformin had developmental defects and either arrested or died and thus adult animals to test for anoxia survival could not be obtained.

The animals fed an OP50 diet (raised at 25°C or 20°C) or animals fed an HT115 diet (raised at 20°C) and supplemented with metformin did not have a higher unimpaired phenotype compared to those not supplemented with metformin (Figure 6A). This data shows that metformin increases anoxia survival but not the unimpaired phenotype. However, the *aak-2* mutant fed the OP50 diet (raised at 20°C) and supplemented with metformin did not survive long-term anoxia. Together, these data provide further evidence that AMPK has a role in inducing an enhanced anoxia survival.

### High carbohydrate levels correlate with enhanced long-term anoxia survival

**Figure 7:**
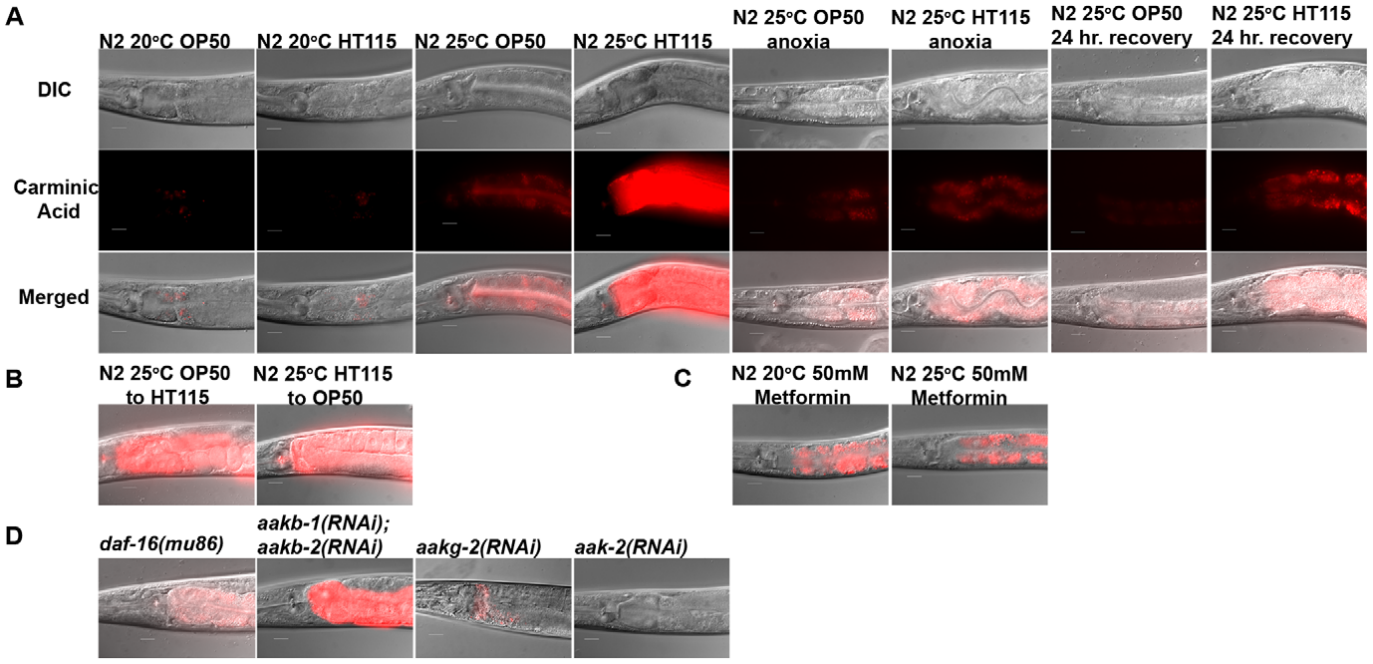
Animals with an enhanced anoxia survival phenotype have increased levels of carminic acid staining. Animals were fed carminic acid, a fluorescent derivative of glucose that incorporates into glycogen and trehalose, to detect carbohydrates in the intestine of *C. elegans*. Images shown include DIC, fluorescent and merged image (A); merged images are show for (B, C). Animals grown at 25°C have increased level of carminic acid which decreases after exposure to long-term anoxia (A). Animals switched to an HT115 diet have an increased level of carminic acid (B). Animals fed metformin, regardless of temperature, have increased level of carminic acid (C). *aak-2(RNAi)* and *aakg-2(RNAi)* suppresses the high level of carminic acid detected in N2 animals raised at 25°C (D). Animals shown are representative of >25 animals assayed for each experiment. Scale bar, 20 µm.

There are several observations correlating glycogen levels with the regulation of AMPK [60], [61]; the β subunits of AMPK contain a glycogen-binding/carbohydrate-binding domain that causes the AMPK complex to bind glycogen in certain conditions [61], [62]. To determine if animals environmentally or genetically preconditioned to survive long-term anoxia have increased levels of carbohydrate stores we used the dye carminic acid. Carminic acid is a fluorescent derivative of glucose that binds to glycogen and trehalose [63], [64]; it has been used in *C. elegans* to detect carbohydrate stores in the intestinal cells of *C. elegans* [65]. We found that wildtype animals grown at 25°C, and fed either OP50 or HT115, had a higher level of carminic acid staining in the intestine than that of wildtype animals raised at 20°C (Figure 7A), suggesting that an increase in temperature physiologically affects the level of carbohydrate stores. Animals exposed to three days of anoxia had a decreased level of carminic acid staining supporting the idea that carbohydrates get utilized during anoxia treatment. Post-anoxia animals that were fed the HT115 diet, in comparison to those fed the OP50 diet, had a higher level of carminic acid staining (Figure 7A). Thus, it is possible that the HT115 diet helps the animals recover from the anoxia treatment.

Animals that were fed OP50 and switched to HT115 diet prior to anoxia treatment had a higher level of carminic acid in comparison to those just fed OP50 (Figure 7B). This further supports the idea that HT115 diet contributes to the level of carbohydrates in the animal. Animals fed the HT115 diet and switched to OP50 did not have a marked decrease in carminic acid (Figure 7B) suggesting that it may take additional time on the OP50 diet to see a decrease in carminic acid. Animals that were fed OP50 supplemented with metformin also had an increased level of carminic acid staining regardless of the growth temperature suggesting that metformin treatment alone can alter carbohydrate stores in *C. elegans* (Figure 7C). We found that RNAi of *aak-2* and *aakg-2* significantly suppressed the levels of carminic acid staining in environmentally preconditioned animals, yet RNAi of *aakb-1*and *aakb-2* did not (Figure 7D). The levels of carminic acid was somewhat reduced in the *daf-16(mu86)* animal (Figure 7D). There were no detectible fluorescence observed in animals not stained with carminic acid indicating that the fluorescence detected in experimental animals fed carminic acid is due to detection of carminic acid and not auto fluorescence (data not shown).

**Figure 8:**
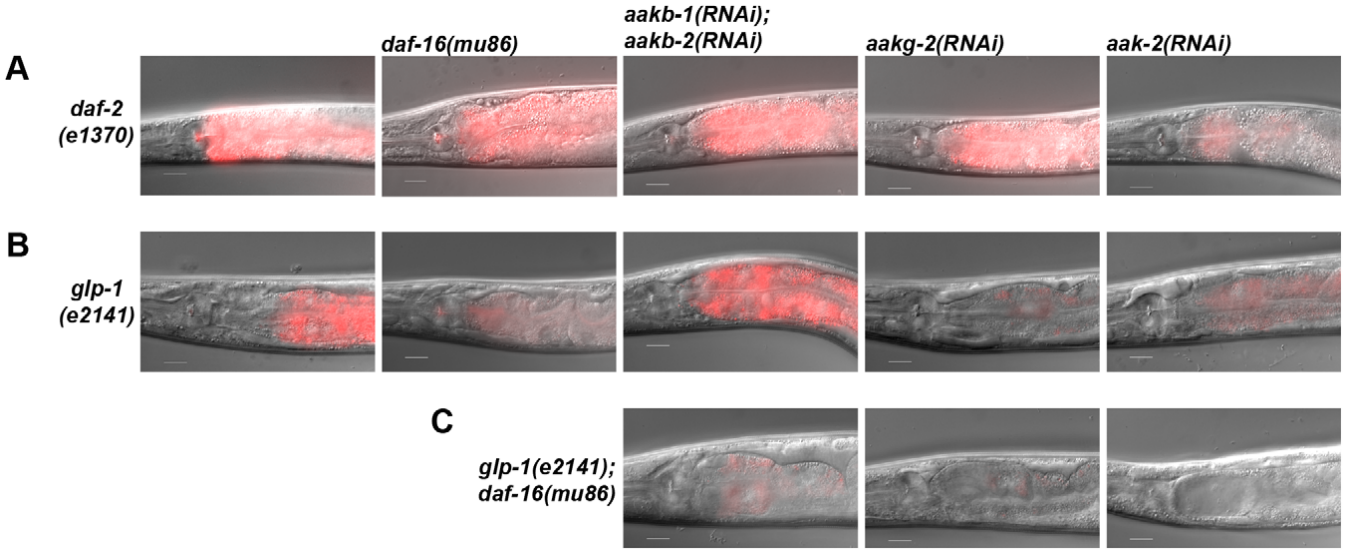
The *daf-2(e1370)* and *glp-1(e2141)* animals have increased levels of carminic acid staining that can be suppressed by specific components of AMPK. Animals of specified genotype or RNAi experiment were fed carminic acid to detect carbohydrate levels in the intestine. Merged images (DIC and fluorescence) of anterior region of the animal are shown. Images shown are representative of >25 number of animals assayed for each experiment. Scale bar, 20 µm.

The *daf-2(e1370)* and *glp-1(e2141)* animals exposed to long-term anoxia have a high survival rate and unimpaired phenotype (Figures 4, 5). Furthermore, the *daf-2(e1370)* and *glp-1(e2141)* animals also have high level of carminic acid (Figure 8). RNAi of *aak-2* suppresses the higher level of carminic acid staining in *daf-2(e1370)* animals (Figure 8A). Note that RNAi of *aak-2* suppressed the unimpaired phenotype in *daf-2(e1370)* animals exposed to three days of anoxia and the survival rate after four days of anoxia exposure. The suppression of carminic acid levels in *glp-1(e2141)* is more complex to interpret because due to the nature of the allele these animals are raised at 25°C thus the temperature and allele may both contribute to increased carminic acid levels. The *daf-16(mu86) mutation*, *aakg-2(RNAi)* or *aak-2(RNAi)* suppresses the higher level of carminic acid staining in *glp-1(e2141)* (Figure 8C). Double RNAi of *aakb-1* and *aakb-2* did not suppress the high levels of carminic acid in *glp-1(e2141)* animals except in the *daf-16(mu86)* background (Figure 8). This suggests that both DAF-16 and AMPK are involved with long-term anoxia survival in *glp-1(e2141)* animals.

## Discussion

With the exception of a few organisms, most multicellular organisms are very sensitive to anoxic environments [66]. Some organisms, such as aquatic turtles, diving seals, brine shrimp, killifish and nematodes can survive bouts of severe oxygen deprivation; these periodically low oxygen levels do not seem to induce detrimental damage to tissues and organs. Nematodes are able to survive anaerobic conditions, in fact the mycophagous nematode *Aphelenchus avenae* were reported to survive 90–95% after exposure to 20 days of anaerobic conditions [67]. *C. elegans* will survive 1–3 days of anoxia depending on the developmental stage; the changes in metabolic processes likely have an affect on viability of animals exposed to anoxia [32], [35], [38]. Arrest of biological processes such as cell division, development and movement is likely a means of surviving anoxia by decreasing energetically requiring processes. Using *C. elegans* it is also becoming increasingly clear that genetic mutations that alter metabolic processes, such as mutations in the insulin-like receptor *daf-2* that alters the function of the FOXO transcription DAF-16, lead to an increase in anoxia survival [38], [68]. Thus genetic changes can alter the physiology of the animal so that survival of severe oxygen deprivation occurs.

It is of interest for human health related issues to identify preconditioning environments; an understanding of such may aid in therapeutic approaches required for recovery from oxygen deprivation or ischemic insult. We found in *C. elegans* that an elevated temperature and HT115 *E. coli* diet precondition for an enhanced long-term anoxia phenotype. This enhanced long-term anoxia phenotype includes a significantly higher survival rate; the survivors have normal movement after long-term anoxia treatment indicating that tissue and organ maintenance occurs. Note that animals raised at 25°C and fed an OP50 *E. coli* diet also have a significantly higher survival rate compared to animals raised at 20°C, but the majority of the survivors have motility defects. Together, our results suggest that synergetic processes may be induced as a result of the two environments (25°C temperature and HT115 *E. coli* food source). The specific mechanism inducing the long-term anoxia survival phenotype is not understood; however, our results indicate that energy stores may be involved. What is not understood is how a slight increase in temperature (20°C to 25°C, which is not typically thought of as a stressful environment for *C. elegans*) or a change in equally accepted laboratory food sources influence an animal's ability to maintain tissue structure and/or function (an unimpaired versus an impaired phenotype) after anoxia treatment. It is possible that these preconditioning environments could alter the expression of specific genes that prepare the animal for stress. Alternatively, it could be that there is an alteration of energy stores, perhaps inducing a dietary restriction-like state for specific macromolecules. For example, lipids are depleted in nematodes grown at 27°C [67] and here we show that carbohydrates stores in the gut are increased in animals grown at 25°C instead of 20°C. Another explanation for our results is that the bacterial strains differ in macronutrients that in turn may influence the metabolism of the worm. For example, it is known that HT115 bacteria have a higher total carbohydrate levels than OP50 [69]; thus it is reasonable to predict that dietary source for *C. elegans* affects metabolism and responses to stress such as anoxia. It remains to be determined specifically how these physiological changes observed in the nematode contribute to the enhanced long-term anoxia phenotype.

Metformin is a drug that has been traditionally used to treat diabetes in humans. The ability for metformin to reduce blood glucose levels is dependent on the protein threonine kinase LKB1; LKB1 activates AMPK. However, neither LKB1 nor AMPK are thought to be the direct target of metformin. AMPK, which is activated by metformin, alters several physiological states including an inhibition of guconeogenesis in hepatocytes [53]. Here we show that there is an increase in carbohydrate stores in the gut of animals fed metformin and that these animals better survive long-term anoxia exposure. It is possible that the metformin-induced increase in carbohydrates stores provides the necessary metabolic or energy state to survive anoxia; perhaps by supplying carbohydrate source used for ATP production. Note that that carbohydrate levels including glycogen levels decrease in nematodes exposed to anoxic environments [35], [67].

Our current and previous studies show that *C. elegans* can be preconditioned to have an enhanced long-term anoxia phenotype by altering the environment in which the animals develop (25°C, HT115 diet) or altering the animal's genotype (*daf-2* or *glp-1* mutants). The genetic mutants *glp-1(e2141)* and *daf-2(e1370)* have a very enhanced long-term anoxia survival phenotype. It is not clear what the similarities and differences are between environmental preconditioning and genetic alleles conductive for anoxia survival. However, a common theme between the two is that specific subunits of AMPK are involved with the enhanced anoxia phenotype. AMPK is a heterotrimeric protein that regulates a variety of pathways involved with metabolism. Our data suggest that *aak-2*, *aakb-1*, *aakb-2* and *aakg-2* are involved with the enhanced anoxia survival phenotype. Of interest is to identify the specific role AMPK subunits have in responding to various metabolic states.

Data presented here indicates that *daf-16* and components of AMPK have a role in anoxia response and survival. If *aak-2* suppressed long-term anoxia response phenotype solely through inactivation of *daf-16* then there would have been no further suppression observed in genetically or environmentally preconditioned animals. Instead we observe that the *aak-2*;*daf-16* double mutant further suppressed the enhanced anoxia survival phenotype observed in the *glp-1* mutant in comparison to suppression by just *aak-2* or *daf-16* alone. These results suggest that *aak-2* and *daf-16* are working in parallel to influence anoxia response and survival in the *glp-1* mutant.

Work by many researchers have shown that aging is regulated by key genes working in a network of signaling pathways and that there is no single gene or pathway that completely controls the aging process. Furthermore, there is overlap between signaling pathways important for longevity and stress responses including oxygen deprivation. It is likely that several pathways regulate the response to oxygen deprivation and that various pathways will be involved with viability and maintenance of tissue function after anoxia exposure. Our work here shows that metabolic processes involving AMPK have a role in anoxia responses.

## Materials and Methods

### Strains

The following genetic strains were obtained from the Caenorhabditis elegans Genetics Center: N2 Bristol (wild-type), *daf-2(e1370), daf-16(mu86), glp-1(e2141), glp-1(e2141);daf-16(mu86), aak-2(gt33), and aak-2(rr48).* The *daf-2(e1370);daf-16(mu86)* mutant was obtained from the Dillin lab. The N2 and mutant strains were cultured on nematode growth media (NGM) plates seeded with *Escherichia coli* (OP50 or HT115 (W3110, rnc+, *TD1-17*::ΔTn*10*)) and raised at 20°C or 25°C as indicated for each experiment [70].

### RNA Interference Assays

We used RNAi, as previously described, to inhibit the subunits of the AMPK [38]. Briefly, synchronized L1 larvae were collected and grown to adulthood on NGM-IPTG plates (200 mg/ml ampicillin, 12.5 mg/ml tetracycline, and 0.5 mg/ml IPTG) seeded with the appropriate *E. coli* strain for RNAi of a specified gene. Experiments in which the animal was fed HT115 food were fed the *E. coli* strain containing the L4440 plasmid with no insert. The *E. coli* HT115 and RNAi strains were obtained from the MRC Geneservice (Cambridge, UK) [71], [72].

### Thermal Preconditioning and Growth Conditions

To precondition the animals prior to anoxia treatment animals were synchronized and raised to adulthood at 25°C. To synchronize, L1 larvae were obtained by collecting embryos from hypochlorite-treated adults; embryos were given 16 hours to hatch in M9 solution on unseeded NGM plates at 20°C. The time animals were raised at 25°C varied depending on the genotype. Strains containing the *glp-1(e2141)* temperature sensitive allele and experimentally matched control were maintained at 15°C to produce progeny. The *glp-1(e2141)* L1 larvae were raised at 15°C for 24 hours on seeded NGM plates, and then transferred to 25°C for an additional 48 hours to develop to young adults. Strains containing the *daf-2(e1370)* temperature sensitive allele and control were grown to adulthood at 20°C because of the 25°C constitutive dauer phenotype. The *daf-2(e1370)* animals were assayed 96 hours after synchronization, whereas additional strains were assayed 72 hours after synchronization. Experiments were conducted on young adults; anatomical markers such as gonad morphology or approximate hours after L4 to adult molt were used to determine the young adult stage of the animal. For all experiments at least four independent experiments were conducted. Animals fed heat-killed bacteria were first grown on live OP50 or HT115 to adulthood and then transferred to heat killed bacteria for 8 hours prior to anoxia treatment. Verification that the heat-killed bacteria were indeed dead was done by replating the bacteria onto LB media and verifying no growth.

### Anoxia Exposure

For all experiments young adults were collected and exposed to anoxia by using anoxia Bio Bags (Becton Dickinson) as previously described [38]. Anoxic conditions, verified using Resazurin indicators (<.001 kPa of O_2_ detection limit), were obtained within one hour. For the purpose of these studies we refer to long-term anoxia as three or four days of anoxia exposure at 20°C. After anoxia treatment worms recovered in air for 24 hours and were assayed and scored as dead (no activity upon prodding with a platinum wire pick) or survivors. The survivors were scored as unimpaired (alive with no detectable defects in morphology, behavior, or motility using a standard stereomicroscope), or impaired (alive but with detectable defects in morphology, behavior, or motility).

### Time Lapse Microscopy Analysis

To demonstrate the unimpaired and impaired phenotype after anoxia treatment, worms on NGM plates were imaged using a Zeiss stereomicroscope and Axiovision Zeiss imaging software. Images were collected at 2-second intervals over a period of atleast 20 seconds. The collected images were imported into QuickTime Pro (Apple Computer) for visualization.

### Carminic Acid Staining

Carminic acid (Cole-Parmer) staining was done as previously described [63], [65]. Briefly, carminic acid was added to sterile LB media for a final concentration of 1 mg/ml. LB media was filter sterilized and cultured with the OP50, HT115 or specified RNAi strain used to seed NGM plates. For all assays synchronized L1 larvae were grown on the carminc acid NGM plates and assayed as one-day old adults. Microscopy analysis was conducted as previously described [38]. Briefly, adult animals were placed on a 2% agarose pad containing 0.2 mM levamisole in M9. Animals were examined using a Zeiss Axioscope fluorescence microscope; image acquisition was done using Axiovision Zeiss and processed using Adobe Photoshop. For all imaging analysis, exposure was identical (100 ms). For each experiment >25 animals were independently analyzed.

### Metformin Exposure

Metformin exposure assays were done as previously described [73]. Briefly, NGM plates containing either 25, 50, 100, 250 or 500 mM of metformin (Sigma) were used for assays. Animals were exposed to metformin as L1 larvae and throughout development to one-day old adults. For all experiments at least four independent experiments were conducted.

### Statistical Analysis

Experimental animal values were compared to control animal values via a Student's one-tailed T-test when comparing two groups. When comparing more than two groups, a one-way ANOVA on ranks was performed. This was followed by an SNK multiple range test or a Dunnett's multiple range test. Alpha levels of 05 were deemed as significant. Analyses were performed in either MS Excel (Microsoft) or SigmaStat software (Systat Software inc.).

## Supporting Information

Video S1 Time-lapse microscopy of wildtype adult hermaphrodite that survived anoxia. Animal was grown on OP50 at 25°C exposed to three days of anoxia and scored as having an impaired phenotype.
(AVI)

Video S2 Time-lapse microscopy of wildtype adult hermaphrodite that survived anoxia. Animal was grown on OP50 at 25°C, exposed to three days of anoxia and scored as having an unimpaired phenotype.
(AVI)

Table S1 Survival rate of environmentally preconditioned wild-type animals exposed to anoxia.
(DOCX)

Table S2 Suppression analysis of environmentally preconditioned animals.
(DOCX)

Table S3 Suppression analysis of long-term anoxia survival in *daf-2(e1370)* animals.
(DOCX)

Table S4 Suppression analysis of long-term anoxia survival in *glp-1(e2141)* animals.
(DOCX)

Table S5 Suppression analysis of long-term anoxia survival in *glp-1(e2141);daf-16(mu86)* animals.
(DOCX)

Table S6 Metformin increases long-term anoxia survival rate of wildtype animals.
(DOCX)
