# Supplementary material for: Environmental and Genetic Preconditioning for Long-Term Anoxia Responses Requires AMPK in Caenorhabditis elegans
Source: PLoS One. 2011 Feb 3;6(2):e16790. doi: 10.1371/journal.pone.0016790 (PMC3033420; doi:10.1371/journal.pone.0016790)
Supplement: Table S1 — Survival rate of environmentally preconditioned wild-type animals exposed to anoxia. (DOCX) [file pone.0016790.s003.docx]

| **Table S1. Survival rate of environmentally preconditioned wild-type animals exposed to anoxia** | | | |
| --- | --- | --- | --- |
| Pre-Anoxia Exposure Environment | |  |  |
| Temperature (°C) | *E. coli* Strain (antibiotics) | Anoxia Exposure (days) | Survival Rate ± SD |
| 20 | OP50 | 3 | 10.4 ±12.0 |
| 25 | OP50 | 3 | 90.1 ±8.6 ^a^ |
| 20 | HT115 | 3 | 3.1 ± 3.6 |
| 25 | HT115 | 3 | 86.5 ±6.8 ^b^ |
| 25 | HT115 (Amp, Tet) | 3 | 90.0 ±6.3 ^b^ |
| 20 | OP50 | 4 | 0.0 ±0.0 |
| 25 | OP50 | 4 | 13.7 ±10.0 |
| 20 | HT115 | 4 | 0.0 ±0.0 |
| 25 | HT115 | 4 | 78.8 ±20.1 ^b^ |
| 25 | HT115 (Amp, Tet) | 4 | 80.8 ±8.1 ^b^ |
| 25 | OP50 | 3 | 85.2 ±17.7 |
| 25 | HT115 | 3 | 95.5 ±1.1 |
| 25 | OP50 to HT115 | 3 | 95.7 ±4.1 |
| 25 | HT115 to OP50 | 3 | 87.0 ±11.4 |
| 25 | OP50 | 4 | 13.0 ±15.4 |
| 25 | HT115 | 4 | 88.8 ±9.9 ^c^ |
| 25 | OP50 to HT115 | 4 | 82.8 ±11.5 ^c^ |
| 25 | HT115 to OP50 | 4 | 47.8 ±41.1 |
| 25 | OP50 to HK OP50 | 3 | 87.4 ±15.9 |
| 25 | OP50 to HK HT115 | 3 | 88.9 ±10.4 |
| 25 | HT115 to HK HT115 | 3 | 89.6 ±5.5 |
| 25 | OP50 to HK HT115 | 3 | 94.2 ±2.6 |
| 25 | OP50 to HK OP50 | 4 | 50.1 ±7.1^c^ |
| 25 | OP50 to HK HT115 | 4 | 52.4 ±3.6^c^ |
| 25 | HT115 to HK HT115 | 4 | 54.5 ±10.4 |
| 25 | OP50 to HK HT115 | 4 | 57.2 ±10.9 |

Survival rates for data presented in Figure 1 and Figure 2

For all experiments N2 strain was used

Bacteria was heat killed (HK) by exposing to 65°C for 30 minutes prior to seeding NGM

^a^ P<.05 in comparison to animals grown on OP50 at 20°C; identical anoxia exposure.

^b^ P<.05 in comparison to animals grown on HT115 at 20°C; identical anoxia exposure.

^c^ P<.05 in comparison to animals grown only on OP50 at 25°C; identical anoxia exposure.
