# Supplementary material for: Environmental and Genetic Preconditioning for Long-Term Anoxia Responses Requires AMPK in Caenorhabditis elegans
Source: PLoS One. 2011 Feb 3;6(2):e16790. doi: 10.1371/journal.pone.0016790 (PMC3033420; doi:10.1371/journal.pone.0016790)
Supplement: Table S2 — Suppression analysis of environmentally preconditioned animals. (DOCX) [file pone.0016790.s004.docx]

| **Table S2. Suppression analysis of environmentally preconditioned animals** | | |
| --- | --- | --- |
| Genotype | Anoxia Exposure (days) | Survival Rate ± SD |
| N2 | 3 | 93.6 ±3.9 |
| *daf-16(mu86)* | 3 | 84.6 ±39.1 |
| *aak-2(rr48)* | 3 | 81.3 ±38.4 |
| *aak-2(gt33)* | 3 | 71.2 ±29.8 |
| *aak-2(RNAi)* | 3 | 75.0 ±13.4 |
| *aak-1(RNAi)* | 3 | 95.5 ±5.2 |
| *aakb-1(RNAi)* | 3 | 97.3 ±1.4 |
| *aakb-2(RNAi)* | 3 | 98.6 ±1.2 |
| *aakg-1(RNAi)* | 3 | 89.6 ±9.8 |
| *aakg-2(RNAi)* | 3 | 82.5 ±4.9 |
| *aakg-4(RNAi)* | 3 | 93.5 ±8.1 |
| *aakg-5(RNAi)* | 3 | 98.7 ±1.1 |
| *aakb-1(RNAi);aakb-2(RNAi)* | 3 | 78.4 ±22.4 |
| *daf-16(mu86);aak-2(RNAi)* | 3 | 48.9 ±31.7 |
|  |  |  |
| N2 | 4 | 78.8 ±10.6 |
| *daf-16(mu86)* | 4 | 56.9 ±12.2 |
| *aak-2(rr48)* | 4 | 11.5 ±16.1 ^a^ |
| *aak-2(gt33)* | 4 | 6.9 ±11.2 ^a^ |
| *aak-2(RNAi)* | 4 | 48.7 ±16.2 |
| *aak-1(RNAi)* | 4 | 90.1 ±8.6 |
| *aakb-1(RNAi)* | 4 | 95.2 ±1.0 |
| *aakb-2(RNAi)* | 4 | 90. ±6.4 |
| *aakg-1(RNAi)* | 4 | 88.1 ±6.3 |
| *aakg-2(RNAi)* | 4 | 41.9 ±40.4 ^a^ |
| *aakg-4(RNAi)* | 4 | 94.8 ±1.6 |
| *aakg-5(RNAi)* | 4 | 98.1 ±.13 |
| *aakb-1(RNAi);aakb-2(RNAi)* | 4 | 44.3 ±10.0 ^a^ |
| *daf-16(mu86);aak-2(RNAi)* | 4 | 16.5 ±16.5 ^a^ |

Survival rates for data presented in Figure 3

For all experiments the *E. coli* food source was HT115 and NGM was supplemented with ampicillin and tetracycline

^a^ P<.05 in comparison to wildtype animals and identical anoxia exposure.
