# Supplementary material for: Environmental and Genetic Preconditioning for Long-Term Anoxia Responses Requires AMPK in Caenorhabditis elegans
Source: PLoS One. 2011 Feb 3;6(2):e16790. doi: 10.1371/journal.pone.0016790 (PMC3033420; doi:10.1371/journal.pone.0016790)
Supplement: Table S3 — Suppression analysis of long-term anoxia survival in daf-2(e1370) animals. (DOCX) [file pone.0016790.s005.docx]

| **Table S3. Suppression analysis of long-term anoxia survival in *daf-2(e1370)* animals** | | |
| --- | --- | --- |
| Genotype | Anoxia Exposure (days) | Survival Rate ± SD |
| *daf-2(e1370)* | 3 | 96.3 ±2.4 |
| *daf-2(e1370);aak-1(RNAi)* | 3 | 96.1 ±3.1 |
| *daf-2(e1370);aak-2(RNAi)* | 3 | 85.9 ±10.6 |
| *daf-2(e1370);aakb-1(RNAi)* | 3 | 97.9 ±2.9 |
| *daf-2(e1370);aakb-2(RNAi)* | 3 | 98.4 ±1.1 |
| *daf-2(e1370);aakg-1(RNAi)* | 3 | 98.9 ±2.1 |
| *daf-2(e1370);aakg-2(RNAi)* | 3 | 97.7 ±3.4 |
| *daf-2(e1370);aakg-4(RNAi)* | 3 | 98.4 ±1.1 |
| *daf-2(e1370);aakg-5(RNAi)* | 3 | 100.0 ±0.0 |
| *daf-2(e1370);aakb-1(RNAi);aakb-2(RNAi)* | 3 | 89.9 ±4.3 |
|  |  |  |
| *daf-2(e1370)* | 4 | 96.4 ±3.9 |
| *daf-2(e1370),aak-1(RNAi)* | 4 | 94.8 ±4.7 |
| *daf-2(e1370);aak-2(RNAi)* | 4 | 68.5 ±15.5 ^a^ |
| *daf-2(e1370);aakb-1(RNAi)* | 4 | 99.3 ±1.3 |
| *daf-2(e1370);aakb-2(RNAi)* | 4 | 100.0 ±0.0 |
| *daf-2(e1370);aakg-1(RNAi)* | 4 | 95.0 ±5.3 |
| *daf-2(e1370),aakg-2(RNAi)* | 4 | 100.0 ±0.0 |
| *daf-2(e1370);aakg-4(RNAi)* | 4 | 97.9 ±2.0 |
| *daf-2(e1370);aakg-5(RNAi)* | 4 | 99.3 ±1.1 |
| *daf-2(e1370),aakb-1(RNAi);aakb-2(RNAi)* | 4 | 86.3 ±10.3 |

Survival rates for data presented in Figure 4

For all experiments the *E. coli* food source was HT115 and NGM was supplemented with ampicillin and tetracycline

Animals were grown at 20°C due to *daf-2(e1370)* dauer constitutive phenotype.

^a^ P<.05 in comparison to *daf-2(e1370)* animals exposed to identical anoxic conditions.
