# Supplementary material for: Environmental and Genetic Preconditioning for Long-Term Anoxia Responses Requires AMPK in Caenorhabditis elegans
Source: PLoS One. 2011 Feb 3;6(2):e16790. doi: 10.1371/journal.pone.0016790 (PMC3033420; doi:10.1371/journal.pone.0016790)
Supplement: Table S4 — Suppression analysis of long-term anoxia survival in glp-1(e2141) animals. (DOCX) [file pone.0016790.s006.docx]

| **Table S4. Suppression analysis of long-term anoxia survival in *glp-1(e2141)* animals.** | | |
| --- | --- | --- |
| Genotype | Anoxia Exposure (days) | Survival Rate ± SD |
| *glp-1(e2141)* | 3 | 100.0 ±0.0 |
| *glp-1(e2141);aak-1(RNAi)* | 3 | 99.5 ±.98 |
| *glp-1(e2141) aak-2(RNAi)* | 3 | 99.0 ±1.9 |
| *glp-1(e2141);aakb-1(RNAi)* | 3 | 100.0 ±0.0 |
| *glp-1(e2141);aakb-2(RNAi)* | 3 | 100.0 ±0.0 |
| *glp-1(e2141);aakg-1(RNAi)* | 3 | 98.7 ±1.2 |
| *glp-1(e2141);aakg-2(RNAi)* | 3 | 98.5 ±1.8 |
| *glp-1(e2141) ;aakg-4(RNAi)* | 3 | 100.0 ±0.0 |
| *glp-1(e2141);aakg-5(RNAi)*  *glp-1(e2141);aakb-1(RNAi);aakb-2(RNAi)* | 3  3 | 100.0 ±0.0  98.9 ±2.3 |
|  |  |  |
| *glp-1(e2141)* | 4 | 100.0 ±0.0 |
| *glp-1(e2141);aak-1(RNAi)* | 4 | 100.0 ±0.0 |
| *glp-1(e2141) aak-2(RNAi)* | 4 | 98.1 ±1.5 |
| *glp-1(e2141);aakb-1(RNAi)* | 4 | 99.3 ±1.2 |
| *glp-1(e2141);aakb-2(RNAi)* | 4 | 97.6 ±2.1 |
| *glp-1(e2141);aakg-1(RNAi)* | 4 | 100.0 ±0.0 |
| *glp-1(e2141);aakg-2(RNAi)* | 4 | 96.4 ±5.7 |
| *glp-1(e2141) ;aakg-4(RNAi)* | 4 | 99.2 ±1.4 |
| *glp-1(e2141);aakg-5(RNAi)*  *glp-1(e2141);aakb-1(RNAi);aakb-2(RNAi)* | 4  4 | 99.7 ±0.7  98.2 ±3.7 |

Survival rates for data presented in Figure 5

For all experiments the *E. coli* food source was HT115 and NGM was supplemented with ampicillin and tetracycline

Due to the *glp-1* sterile phenotype L1 larvae were grown at 15°C for 24 hours and then transferred to 25°C and allowed to develop to young adults; all controls were grown in an identical manner.
