# Supplementary material for: Environmental and Genetic Preconditioning for Long-Term Anoxia Responses Requires AMPK in Caenorhabditis elegans
Source: PLoS One. 2011 Feb 3;6(2):e16790. doi: 10.1371/journal.pone.0016790 (PMC3033420; doi:10.1371/journal.pone.0016790)
Supplement: Table S5 — Suppression analysis of long-term anoxia survival in glp-1(e2141);daf-16(mu86) animals. (DOCX) [file pone.0016790.s007.docx]

| **Table S5. Suppression analysis of long-term anoxia survival in**  ***glp-1(e2141);daf-16(mu86)* animals.** | | |
| --- | --- | --- |
| Genotype | Anoxia Exposure (days) | Survival Rate ± SD |
| *glp-1(e2141);daf-16(mu86)* | 3 | 100.0 ±0.0 |
| *glp-1(e2141); daf-16(mu86);aak-1(RNAi)* | 3 | 100.0 ±0.0 |
| *glp-1(e2141);daf-16(mu86);aak-2(RNAi)* | 3 | 93.3 ±3.8 |
| *glp-1(e2141);daf-16(mu86);aakb-1(RNAi)* | 3 | 97.5 ±.89 |
| *glp-1(e2141);daf-16(mu86);aakb-2(RNAi)* | 3 | 96.7 ±1.1 |
| *glp-1(e2141);daf-16(mu86);aakg-1(RNAi)* | 3 | 97.2 ±3.0 |
| *glp-1(e2141);daf-16(mu86);aakg-2(RNAi)* | 3 | 94.9 ±2.5 |
| *glp-1(e2141);daf-16(mu86);aakg-4(RNAi)* | 3 | 95.5 ±4.9 |
| *glp-1(e2141); daf-16(mu86);aakg-5(RNAi)* | 3 | 98.8 ±2.0 |
| *glp-1(e2141),daf-16(mu86);aakb-1(RNAi);aakb-2(RNAi)* | 3 | 99.5 ±1.1 |
|  |  |  |
| *glp-1(e2141),daf-16(mu86)* | 4 | 95.8 ±3.0 |
| *glp-1(e2141); daf-16(mu86);aak-1(RNAi)* | 4 | 95.0 ±6.4 |
| *glp-1(e2141),daf-16(mu86);aak-2(RNAi)* | 4 | 61.3 ±9.7 ^a^ |
| *glp-1(e2141);daf-16(mu86);aakb-1(RNAi)* | 4 | 97.1 ±5.0 |
| *glp-1(e2141);daf-16(mu86);aakb-2(RNAi)* | 4 | 97.4 ±1.0 |
| *glp-1(e2141);daf-16(mu86);aakg-1(RNAi)* | 4 | 94.3 ±3.7 |
| *glp-1(e2141),daf-16(mu86);aakg-2(RNAi)* | 4 | 71.3 ±11.2 ^a^ |
| *glp-1(e2141);daf-16(mu86);aakg-4(RNAi)* | 4 | 97.4 ±2.8 |
| *glp-1(e2141); daf-16(mu86);aakg-5(RNAi)* | 4 | 98.2 ±1.5 |
| *glp-1(e2141),daf-16(mu86);aakb-1(RNAi);aakb-2(RNAi)* | 4 | 74.0 ±29.9 ^a^ |

Survival rates for data presented in Figure 5

For all experiments the *E. coli* food source was HT115 and NGM was supplemented with ampicillin and tetracycline

Due to the *glp-1* sterile phenotype L1 larvae were grown at 15°C for 24 hours and then transferred to 25°C and allowed to develop to young adults; all controls were grown in an identical manner.

^a^P<.05 in comparison to *glp-1(e2141)* animals exposed to identical anoxic conditions.
