# Supplementary material for: Environmental and Genetic Preconditioning for Long-Term Anoxia Responses Requires AMPK in Caenorhabditis elegans
Source: PLoS One. 2011 Feb 3;6(2):e16790. doi: 10.1371/journal.pone.0016790 (PMC3033420; doi:10.1371/journal.pone.0016790)
Supplement: Table S6 — Metformin increases long-term anoxia survival rate of wildtype animals. (DOCX) [file pone.0016790.s008.docx]

| **Supplementary Table 6. Metformin increases long-term anoxia survival rate of wildtype animals** | | | |
| --- | --- | --- | --- |
| Strain | Media | Development  Temperature (^o^C) | Survival Rate ± SD |
| N2 | OP50 control | 20 | 5.1 ±4.9 |
| N2 | OP50 | 25 | 90.5 ±5.2 |
| N2 | OP50 + 25mM Metformin | 20 | 48.2 ±40.1 |
| N2 | OP50 + 50mM Metformin | 20 | 82.0 ±4.0^a^ |
| N2 | OP50 + 100mM Metformin | 20 | 87.1 ±9.95 |
| N2 | OP50 + 50mM Metformin | 25 | 92.8 ±7.8 |
| N2 | HT115 + 50mM Metformin | 20 | 94.3 ±5.1 |
| *aak-2(gt33)* | OP50 + 50mM Metformin | 20 | 20.4 ±4.5^b^ |

Survival rates for data presented in Figure 6

^a^ P<.05 in comparison to N2 grown on OP50 at 20^o^C

^b^ P<.05 in comparison to N2 on OP50 + 50mM Metformin at 20^o^C
